# Hearing Loss and Quality of Life (QOL) among Human Immunodeficiency Virus (HIV)-Infected and Uninfected Adults

**DOI:** 10.4172/2155-6113.1000645

**Published:** 2016-12-19

**Authors:** N Duong, P Torre, G Springer, C Cox, MW Plankey

**Affiliations:** 1 School of Medicine and Health Sciences, George Washington University, Washington, District of Columbia, USA; 2 School of Speech, Language, and Hearing Sciences, San Diego State University, San Diego, California, USA; 3 Department of Epidemiology, Johns Hopkins University Bloomberg School of Public Health, Baltimore, Maryland, USA; 4 Department of Medicine, Division of Infectious Diseases, Georgetown University Medical Center, Washington, District of Columbia, USA

**Keywords:** Multicenter AIDS cohort study, Women's interagency HIV study, Auditory impairment, QOL

## Abstract

**Objective:**

Research has established that human immunodeficiency virus (HIV) causes hearing loss. Studies have yet to evaluate the impact on quality of life (QOL). This project evaluates the effect of hearing loss on QOL by HIV status.

**Methods:**

The study participants were from the Multicenter AIDS Cohort Study (MACS) and the Women's Interagency HIV study (WIHS). A total of 248 men and 127 women participated. Pure-tone air conduction thresholds were collected for each ear at frequencies from 250 through 8000 Hz. Pure-tone averages (PTAs) for each ear were calculated as the mean of air conduction thresholds in low frequencies (i.e., 250, 500, 1000 and 2000 Hz) and high frequencies (i.e., 3000, 4000, 6000 and 8000 Hz). QOL data were gathered with the Short Form 36 Health Survey and Medical Outcome Study (MOS)-HIV instrument in the MACS and WIHS, respectively. A median regression analysis was performed to test the association of PTAs with QOL by HIV status.

**Results:**

There was no significant association between hearing loss and QOL scores at low and high pure tone averages in HIV positive and negative individuals. HIV status, HIV biomarkers and treatment did not change the lack of association of low and high pure tone averages with poorer QOL.

**Conclusion:**

Although we did not find a statistically significant association of hearing loss with QOL by HIV status, testing for hearing loss with aging and recommending treatment may offset any presumed later life decline in QOL.

## Introduction

Presbycusis, bilateral insidious hearing loss and cochlear dysfunction, is a degenerative process characterized by three key elements: deterioration of auditory sensitivity, loss of sensory cells, and loss of central auditory processing functions [1,2]. It is one of the most prevalent chronic conditions, and is the most common sensory modality loss in the elderly population. It is estimated that over 25-40% of those over 65 year experience some form of hearing impairment [2-4]. Hearing loss has been found to decrease quality of life (QOL) measures via different mechanisms. Basically it compromises communication and thus one's social function [2,5-13]. Individuals with hearing loss are more susceptible to experiencing mental and physical health decline. Currently, there is a lack of resources and awareness for hearing loss, which poses a large public health burden [9].

Otologic symptoms and conditions have been reported in patients with human immunodeficiency virus infection and acquired immune deficiency syndrome (HIV/AIDS) [14-22]. Shouten et al. investigated the association of antiretroviral therapy using zidovudine (AZT) or didanosine (ddI) with hearing sensitivity [23]. There were no statistically significant changes in either high or low pure tone average frequencies, after accounting for age, noise exposure, CD4+ cell count, and HIV RNA viral load. van der Westhuizen et al. found that age, gender and race matched HIV-infected adults had an approximately 34% higher rate of hearing loss (HL) using pure tone averages of 0.5, 1 and 2 kHz >25 decibels (dB) compared to controls (approximately 4%) [24]. HIV-infected adults also had higher pure-tone thresholds across all of the frequencies measured (0.5, 1, 2, 3 and 4 kHz) and significantly higher rate of sensorineural hearing loss in those adults with CDC Class C disease progression status. Luque et al. found no significant difference in hearing loss (defined as four-frequency (0.5, 1, 2 and 4 kHz) pure tone averages >25 dB HL in either ear) among HIV-infected compared to uninfected adults [25]. This loss among the HIV-infected adults was independent of HIV duration, HIV RNA viral load, and baseline and nadir CD4+ cell count. However, the prevalence of hearing loss was 18.9% with late stage HIV disease. Maro et al. study of 449 HIV-infected and 202 HIV-uninfected adults [26] found no significant differences in hearing sensitivity thresholds for either ear adjusted for age, sex, and noise exposure and whether they were taking antiretroviral medications or not. Lastly, Torre and our group [27] analyzed hearing data from the Multicenter AIDS Cohort Study (MACS) [28] and Women's Interagency HIV study (WIHS) [29]. We found 12% poorer low (250 to 2000 Hz) and 18% poorer high (3000 to 8000 Hz) frequency hearing among 117 HIV-infected men and 105 women as compared to 174 HIV-uninfected adults. HIV disease biomarkers and anti-retroviral treatment history did not explain these differences. In addition to studies investigating the relationship of peripheral hearing loss and HIV infection, studies have shown that HIV affects the central audiotory system [30-34], which are consistent with other HIV-related central nervous system disorders [35,36].

QOL measures reflect disease burden, monitor changes in health, and track treatment efficacy. It is a multi-dimensional concept that includes psychosocial, psychological, physical and mental well-being [5,6,8,37,38]. A study by Wong et al. found that the Chinese perceive hearing loss as a natural part of aging and that they isolate themselves irrespective of the presence or absence hearing loss [38]. Nonetheless, the hearing impaired demonstrated an overall worse general and hearing specific QOL. One study found that bilateral, combined high and low frequency loss had the most significant impact on QOL [5]. In another study people with idiopathic sudden sensorineural hearing loss (ISHL) and bilateral sensorineural hearing loss had worse QOL measures than that of average Japanese patients except for bodily pain and vitality scores [39].

Hearing loss has been shown to be independently associated with social isolation [40-42], which can adversely impact QOL among older adults [43]. Social isolation can be driven by stigma associated with marginalizing chronic medical conditions such as HIV [44]. In a study of a Veterans Affairs population with and without HIV, Greysen et al. [21] found that social isolation was a risk factor for increased rates of hospitalization and mortality irrespective of HIV status. However, they also demonstrated that being HIV+ as well as increasing age was associated with higher self-reported isolation scores. [45]. Therefore we conceptualized the association between objective clinical outcomes (such as hearing loss in this study) and subjective patient experiences (such as QOL) grounded in the overarching relationships of hearing loss and HIV, social isolation and HIV disease and social isolation and hearing loss in this work [46,47].

We are not aware of any published literature that has investigated the relationship between hearing loss and QOL and whether if differs by HIV status using used data collected from the MACS and WIHS. The primary hypothesis for this study was that being hearing impaired negatively impacts QOL, and this effect is moderated by HIV status.

## Materials and Methods

The institutional review boards from San Diego State University, Johns Hopkins Bloomberg School of Public Health, Georgetown University, and Whitman-Walker Health approved this study. Written informed consent was obtained from all study participants.

### Participants and procedures

Participants from the Baltimore-Washington, DC site of the MACS and the Washington, DC site of the WIHS, ongoing prospective observational cohorts investigating the progression of HIV infection, comprised the study sample. The MACS consists of over 7000 HIV+ and HIV- men who have sex with men, beginning in 1984 to 1985 at 4 centers located in Baltimore, MD/Washington, D.C., Chicago, IL, Los Angeles, CA and Pittsburgh, PA. Similarly, the WIHS consists of a cohort of 3,766 women (2,791 HIV-infected and 975 HIV-uninfected) were enrolled in either 1994-1995 (n=2,623) or 2001-2002 (n=1,143) from six United States cities [New York, NY, Chicago, IL, Los Angeles, CA, San Francisco, CA and Washington, D.C.]. Every six months, both MACS and WIHS participants complete a comprehensive physical examination, provide blood specimens for CD4+, CD8+ T-cell count and HIV-RNA determination and complete an interviewer-administered questionnaire, which provided data regarding socio-demographics and medical history including antiretroviral therapy use. The MACS and WIHS uses a standard definition of HAART adapted from the Department of Health and Human Services/Kaiser Panel guidelines [48]. Specific details regarding the MACS and WIHS study design and recruitment are outlined elsewhere [29].

The participants completed a clinical hearing examination consisting of an otoscopic examination, tympanometry, and pure-tone air and bone conduction testing. Full details of the testing protocol are outlined elsewhere [27].

### Primary predictor variables

Pure-tone bone and air conduction testing results were used to assess the participants' hearing loss. Pure-tone air conduction thresholds were measured in each ear at 250, 500, 1000, 2000, 3000, 4000, 6000 and 8000 Hz. Pure-tone averages (PTAs) for each ear were calculated as the mean of air conduction thresholds in low frequencies (LPTA: i.e., 250, 500, 1000 and 2000 Hz) and high frequencies (HPTA: i.e., 3000, 4000, 6000 and 8000 Hz), respectively. Participant's lowest HPTA and LPTA were used as the primary predictor variables.

### Covariates

Participant's age, race/ethnicity (black vs. non-black), and HIV status were investigated as covariates. For the sub-analyses among the HIV+ participants only, CD4+ T-cell count, CD8+ T-cell count, ever being diagnosed with clinical AIDS, log_10_ HIV RNA and cumulative duration (years) of antiretroviral therapy (ART) use classified as non-nucleotide/nucleoside reverse transcriptase inhibitor (NNRTI), nucleotide/nucleoside reverse transcriptase inhibitor (NRTI) and protease inhibitor (PI).

Any AIDS-defining illnesses including a history of pulmonary tuberculosis were self-reported according to the 1993 CDC definition of AIDS. Cumulative duration (years) of use of each class of ART was calculated on the basis of the number of ART medications reported in each classification and weighted for self-reported adherence. Weights were calculated by multiplying the number of ART medications at each visit by the adherence level, and the weighted values then cumulated. The weights were 1, 0.975, 0.85, 0.375 and 0 for adherence levels of 100%, 95% to 99%, 75% to 94%, less than 75% and 0%, respectively. ART use prior to October 1998 was considered 100% adherent.

In the MACS, plasma HIV RNA concentrations were measured using the COBAS Ultrasensitive Amplicor HIV-1 monitor assay (Roche Molecular Systems), sensitive to 50 copies HIV RNA/mL; or the Taqman HIV-1 Test (Roche Molecular Systems), sensitive to 20 copies HIV RNA/mL. In the WIHS, plasma HIV RNA was measured using the COBAS AmpliPrep/COBAS TaqMan HIV-1 Test (Roche Molecular Systems), sensitive to 20 or 48 copies HIV RNA/mL. Values were log10 transformed for statistical analysis. CD4+ and CD8+ T-cell counts were measured for HIV+ men and women at each study visit using standardized flow cytometry and a complete blood cell count. Laboratory results (CD4+, CD8+, HIV RNA) collected within 1 year prior to the hearing testing date hearing testing date were used for this analysis.

### Outcome variable

Participants' QOL score during the 18 months following the hearing testing was the outcome variable of interest. Higher scores indicate better QOL and function. In the MACS, QOL data were collected using the Short Form-36 Health Survey (SF-36) annually beginning in April 1994, which consists of 8 domains. The physical component score and the mental component scores were calculated following Ware's method [49] and normalized to a mean of 50 +/- 10 [50]. Both physical and mental QOL scores were investigated for the male participants. For the women in the WIHS, annual QOL data were collected using a shortened version of the Medical Outcome Study (MOS)-HIV instrument developed by Bozzette et al. beginning in October 1998 [51]. The shortened version has 21 items representing 9 domains that include: physical functioning, role functioning, energy/fatigue, social functioning, cognitive functioning, pain, emotional well-being, perceived health index and current health perception. The score for each domain is calculated by averaging the raw scores for each corresponding item based on a 0-100 scale with higher scores representing better physical, mental and social functioning. A summary score is calculated using the scores from 6 domains (physical functioning, role functioning, energy/fatigue, social functioning, pain and emotional well-being) based on an established algorithm [52]. The summary QOL index was used for the female participants.

### Statistical analysis

The QOL data were analyzed for each cohort separately due to the different QOL measuring methods. Because of the skewed distribution of the residuals in the PTA data when analyzing QOL measurements, a median regression analysis was performed on the untransformed data with bootstrap standard errors and confidence intervals. A total of 2000 bootstrap samples were selected for each model, and confidence intervals were based on the percentile method. In the model with both HIV+ and HIV- participants, the covariates included age, race/ethnicity, HIV status, lowest LPTA and HPTA. Separate models were constructed to examine QOL data for HIV+ participants only. For models restricted to the HIV+ participants, the covariates were CD4+ T-cell counts, CD8+ T-cells, ever AIDS, log_10_ HIV RNA and cumulative years of ART use, besides age and race/ethnicity. Regression models were performed using the SAS procedure QUANTREG, Version 9.3.

## Results

Among the 375 participants (213 HIV+, 57%) who had a QOL measurement within 18 months after pure-tone audiometry testing, there were 248 men with the average age of 56.9 years old (SD=8.8), of whom 112 (45%) were HIV+ and 127 women with a mean age of 47.9 (SD=8.4), of whom 101 (80%) were HIV+. The characteristics of the study participants are summarized by HIV status and gender in Table 1. Compared to the HIV- participants, the HIV+ participants with higher proportions of female and black race/ethnicity were younger and had similar values of the lowest PTAs in low frequencies but lower values in high frequencies. Among the HIV+ participants, men had a longer cumulative duration of NRTI and NNRTI therapy but a shorter duration of PI therapy compared with women; the HIV+ men had higher CD4+ and CD8+ cell counts; and the HIV+ women had a higher proportion of clinical AIDS.

The results of the multivariable analyses are shown in Tables 2-4. Consistent with our primary hypothesis of effect modification, our initial models included two-way interactions between hearing loss and HIV status. None of these interactions were significant, (data not shown) however, and we therefore present results for main effect models without interaction terms. For men, there was no statistically significant association of HPTA and LPTA after adjusting for age, race/ethnicity and HIV status. For women, there was a borderline negatively statistically significant association with LPTA (*p*=0.07) only and QOL index. For both men and women, the relationship of hearing loss and QOL did not differ by HIV status.

Tables 5-7 show the results of the multivariable analyses restricted to the HIV+ participants only. There were no statistically significant associations of age, race/ethnicity and HIV status including ART use, virologic and immunologic markers with both HPTA and LPTA loss and men's physical/mental QOL score and women's summary QOL index, except that ever AIDS was negatively associated with higher women's QOL index (*p*=0.03).

## Conclusion

Hearing loss affects multiple forms of social, emotional and physical function [9]. The literature reports its association with mood disorders, increased risks of falls and hospitalizations, early mortality and even a rapid deterioration in cognitive function [9]. One study found that HIV+ individuals have poorer hearing across all frequencies as compared to HIV- individuals [27]. This present study is an extension of that knowledge. In this study, we sought to determine if hearing loss in HIV+ and HIV- people impacts QOL differentially at low and high frequencies. In this study, we did not find these relationships in HIV+ individuals.

There are a limited number of studies in the literature that look at hearing loss and its impact on QOL, and none to our knowledge, that have attempted to examine the effect that HIV status has on this relationship. The few studies that do exist have used self-reported hearing loss in evaluating its impact on QOL [41,53-55] and they have found that higher frequency loss was associated with a feeling of social limitation and poorer emotional well-being. Most have had small sample sizes or were cross-sectional in nature, thus causality could not be inferred and the results could have been a result of other comorbidities. However, an advantage of self-report is that subtle forms of impairment could potentially be identified and it is also more representative of function in everyday life. There are a few studies that used clinical assessment of hearing loss to study its impact on QOL measures. Some studies used PTA to define hearing loss [56-58] while others used a free field voice test [59,60]. All found a negative impact of loss on social and emotional function, but Thomas et al. reported that hearing deficits were not associated with emotional status or social integration. One study established that measured hearing loss was associated with higher risk of mortality in men and this effect was moderated by mood levels [59]. To our knowledge, there has been only one population-based study to date that used clinical audiometric data. Dalton et al. [8] found that communication difficulties, as assessed by a hearing handicap survey, communication questionnaire, and audiometry testing were significantly associated with impaired functions of daily living as measured by the SF-36 in older adults. The participants in the Dalton study were much older (average age 69 years) than those in the MACS and WIHS, so that age related hearing loss (both measured and self-reported) is likely to be more prevalent and therefore to impact QOL.

The limitations of this present study include a relatively small sample size and limited variability of QOL measurements. Additionally QOL was not measured longitudinally. Although this may not be the first study that uses clinical assessment of hearing loss, this is the first study to examine the impact of hearing loss on QOL in HIV disease. Given our previous published findings [27] demonstrating higher risk of hearing loss among this sample of HIV+ men and women, our ability to detect poorer QOL may be temporally premature given the sample is middle-aged. Health care providers should still be more cognizant in general of the need to test for hearing loss with aging and recommend treatment in order to identify hearing loss as early as possible to offset the possible decline in QOL later in life.

## Figures and Tables

**Table 1:** Characteristics of participants stratified by HIV status.

	HIV+			HIV-		
	Men	Women	All HIV+	Men	Women	All HIV-
**N**	112	101	213	136	26	162
Age*, **mean (SD), years**	53.7 (7.7)	48.8 (7.7)	51.4 (8.0)	59.4 (8.8)	44.5 (10.3)	57.0 (10.6)
Race*, **n (%)**						
** Non-black**	53 (47.3)	16 (15.8)	69 (32.4)	111 (81.6)	8 (30.8)	119 (73.5)
** Black**	59 (52.7)	85 (84.2)	144 (67.6)	25 (18.4)	18 (69.2)	43 (26.5)
**Ever AIDS, n (%)**	19 (17.0)	40 (39.6)	59 (27.7)			
**Total years spent on NRTI, median (IQR)**	22.13 (12.77, 31.29)	19.82 (9.67, 26.56)	21.52 (11.78, 28.28)			
**Total years spent on NNRTI, median (IQR)**	5.30 (0.72, 9.73)	1.90 (0, 3.95)	2.60 (0, 7.72)			
**Total years spent on PI, median (IQR)**	4.07 (0, 10.57)	7.10 (0, 13.48)	5.41 (0, 12.19)			
**Current CD4+ cell count cells/μl, mean (SD)**	615.7 (293.7)	549.9 (260.8)	584.0 (279.7)			
**Current CD8+ cell count cells/μl, mean (SD)**	887.8 (420.8)	817.1 (425.0)	853.8 (423.3)			
**Log_10_ (HIV RNA, copies/ml), median (IQR)**	1.6 (1.0, 1.6)	1.7 (1.3, 2.8)	1.6 (1.3, 1.7)			
**Lowest LPTA median (IQR)**	13.75 (10.00, 18.13)	10.00 (6.25 16.25)	12.50 (8.75, 17.50)	13.75 (10.00, 18.75)	7.50 (5.00, 12.50)	12.50 (8.75, 17.50)
**Lowest HPTA, median (IQR)**	19.38 (12.50, 31.88)	13.75 (10.00, 21.25)	16.25 (11.25, 26.25)	22.50 (14.38, 34.38)	10.63 (6.25, 13.75)	20.00 (12.50, 33.75)
**QOL score, median (IQR)**						
QOL index*		77.68 (62.68, 85.57)			84.49 (71.58, 92.58)	
Mental health*	53.07 (44.58, 58.35)			56.40 (50.04, 59.48)		
** Physical health**	51.64 (40.54, 55.53)			53.17 (47.82, 56.37)		

HRT: Hormone Replacement Therapy; Ever AIDS: Ever diagnosed with AIDS; NRTI: Nucleoside Analog Reverse Transcriptase Inhibitor; NNRTI: Non-NRTI; PI: Protease Inhibitor; IQR = Interquartile range; log_10_ (HIV RNA): Current log Level of HIV RNA; SD: Standard Deviation; LPTA: Low Frequency Pure-Tone Average; HPTA: High Frequency Pure-Tone Average;

* Comparison (HIV+ vs. HIV-) statistically significant, p-value <0.03

**Table 2:** Estimates of each effect for women's QOL index.

Effect	Estimate	Confidence Intervals	*p*
**Age, 10-yr increase**	-2.53	(-7.67, 2.25)	0.29
**Black**	5.91	(-9.35, 15.26)	0.67
**HIV+**	-3.43	(-12.24, 6.50)	0.48
**Lowest HPTA**	-0.16	(-0.73, 0.67)	0.98
**Lowest LPTA**	-0.70	(-1.83, 0.05)	0.07

LPTA: Low Frequency Pure-Tone Average; HPTA: High Frequency Pure-Tone Average; QOL: Quality Of Life

**Table 3:** Estimates of each effect for men's physical health QOL score.

Effect	Estimate	Confidence Intervals	*p*
**Age, 10 year increase**	-1.21	(-3.22, 0.48)	0.18
**Black**	-2.97	(-7.07, 0.65)	0.12
**HIV+**	-1.34	(-5.22, 0.66)	0.23
**Lowest HPTA**	-0.02	(-0.14, 0.09)	0.64
**Lowest LPTA**	-0.11	(-0.39, 0.20)	0.61

LPTA: Low Frequency Pure-Tone Average; HPTA: High Frequency Pure-Tone Average; QOL: Quality Of Life

**Table 4:** Estimates of each effect for men's mental health QOL score.

Effect	Estimate	Confidence Intervals	*p*
**Age, 10 year increase**	3.63	(1.61, 4.99)	<0.001
**Black**	1.06	(-2.35, 3.26)	0.51
**HIV+**	-2.06	(-4.23, 0.79)	0.16
**Lowest HPTA**	-0.02	(-0.11, 0.10)	0.70
**Lowest LPTA**	-0.03	(-0.32, 0.17)	0.76

LPTA: Low Frequency Pure-Tone Average; HPTA: High frequency pure-tone average; QOL: Quality Of Life

**Table 5:** Estimates of each effect for HIV+ women's QOL index.

Effect	Estimate	Confidence Intervals	*p*
**Age, 10 year increase**	-2.73	(-9.39, 2.60)	0.24
**Black**	7.77	(-4.57, 18.49)	0.22
**CD4+ cell count-100 increase**	1.36	(-0.87, 3.26)	0.19
**CD8+ cell count-100 increase**	-0.04	(-0.88, 1.19)	0.80
**Ever AIDS**	-11.97	(-23.29, -1.35)	0.03
**Log_10_ (HIV RNA, copies/ml)**	-1.51	(-5.93, 2.53)	0.48
**Total years spent on NNRTI**	0.32	(-1.32, 1.86)	0.82
**Total years spent on NRTI**	-0.40	(-0.90, 0.41)	0.37
**Total years spent on PI**	0.43	(-0.44, 1.21)	0.37
**Lowest HPTA**	0.04	(-0.72, 0.70)	0.83
**Lowest LPTA**	-0.24	(-1.64, 0.70)	0.59

LPTA: Low Frequency Pure-Tone Average; HPTA: High frequency pure-tone average; QOL: Quality Of Life

**Table 6:** Estimates of each effect for HIV+ men's physical health QOL score.

Effect	Estimate	Confidence Intervals	*p*
**Age, 10 year increase**	-0.66	(-5.86, 3.29)	0.58
**Black**	-1.04	(-8.14, 4.86)	0.53
**CD4+ cell count, 100 increase**	-0.34	(-1.42, 0.96)	0.60
**CD8+ cell count, 100 increase**	0.42	(-0.54, 0.92)	0.57
**Ever AIDS**	-5.69	(-12.43, 4.69)	0.45
**Log_10_ (HIV RNA, copies/ml)**	-2.29	(-5.73, 1.61)	0.14
**Total years spent on NNRTI**	0.33	(-0.62, 0.94)	0.48
**Total years spent on NRTI**	0.08	(-0.16, 0.48)	0.35
**Total years spent on PI**	-0.10	(-0.69, 0.22)	0.30
**Lowest HPTA**	-0.09	(-0.31, 0.25)	0.74
**Lowest LPTA**	0.18	(-0.58, 0.63)	0.68

LPTA: Low Frequency Pure-Tone Average; HPTA: High Frequency Pure-Tone Average; QOL: Quality Of Life

**Table 7:** Estimates of each effect for HIV+ men's mental health QOL score.

Effect	Estimate	Confidence Intervals	*p*
**Age, 10 year increase**	3.03	(-0.65, 6.92)	0.09
**Black**	4.03	(-4.85, 7.34)	0.47
**CD4+ cell count, 100 increase**	0.06	(-1.15, 0.74)	0.67
**CD8+ cell count, 100 increase**	-0.19	(-0.73, 0.54)	0.70
**Ever AIDS**	-0.66	(-12.84, 3.24)	0.47
**Log_10_ (HIV RNA, copies/ml)**	-1.55	(-2.99,2.42)	0.42
**Total years spent on NNRTI**	0.25	(-0.60, 0.94)	0.64
**Total years spent on NRTI**	0.05	(-0.27, 0.38)	0.60
**Total years spent on PI**	0.04	(-0.23, 0.73)	0.80
**Lowest HPTA**	0.11	(-0.27, 0.31)	0.80
**Lowest LPTA**	-0.26	(-0.69, 0.37)	0.75

LPTA: Low Frequency Pure-Tone Average; HPTA: High Frequency Pure-Tone Average; QOL: Quality Of Life
